# Improving the healthcare response to domestic violence and abuse in primary care: protocol for a mixed method evaluation of the implementation of a complex intervention

**DOI:** 10.1186/s12889-018-5865-z

**Published:** 2018-08-03

**Authors:** Alex Hardip Sohal, Gene Feder, Estela Barbosa, Lee Beresford, Anna Dowrick, Farah El-Shogri, Annie Howell, Natalia Lewis, Medina Johnson, Claire Nightingale, Kambiz Boomla, Stephen Morris, Sandra Eldridge, Chris Griffiths

**Affiliations:** 1 Queen Mary University of London, Centre for Primary Care and Public Health, Barts and The London School of Medicine and Dentistry, London, England; 2 School of Social and Community Medicine, University of Bristol, Bristol, England; 3 Department of Applied Health Research, University College London, London, England; 4 IRISi, Bristol, England

**Keywords:** Domestic violence abuse complex intervention implementation evaluation

## Abstract

**Background:**

Domestic violence and abuse remains a major health concern. It is unknown whether the improved healthcare response to domestic violence and abuse demonstrated in a cluster randomised controlled trial of IRIS (*I*dentification and *R*eferral to *I*mprove *S*afety), a complex intervention, including general practice based training, support and referral programme, can be achieved outside a trial setting. Aim: To evaluate the impact over four years of a system wide implementation of IRIS, sequentially into multiple areas, outside the setting of a trial.

**Methods:**

An interrupted time series analysis of referrals received by domestic violence and abuse workers from 201 general practices, in five northeast London boroughs; alongside a mixed methods process evaluation and qualitative analysis. Segmented regression interrupted time series analysis to estimate impact of the IRIS intervention over a 53-month period. A secondary analysis compares the segmented regression analysis in each of the four implementation boroughs, with a fifth comparator borough.

**Discussion:**

This is the first interrupted time series analysis of an intervention to improve the health care response to domestic violence. The findings will characterise the impact of IRIS implementation outside a trial setting and its suitability for national implementation in the United Kingdom.

**Electronic supplementary material:**

The online version of this article (10.1186/s12889-018-5865-z) contains supplementary material, which is available to authorized users.

## Background

This paper reports the protocol for a system wide implementation evaluation of IRIS - *I*dentification and *R*eferral to *I*mprove *S*afety of women affected by domestic violence and abuse (DVA), a complex intervention, designed to improve the primary healthcare response to DVA.

According to World Health Organization (WHO) and National Institute for Health and Care Excellence (NICE) guidelines, health professionals should be trained to provide assistance for women affected by DVA by facilitating disclosure, checking their safety, offering support and referral, and providing the appropriate medical services and follow-up care [1, 2]. These guideline recommendations are based on research from multiple health settings. This research includes how to effectively identify those affected by DVA and record DVA safely, in emergency care [3], antenatal [4], maternity & sexual health services [5], HIV clinics [6], community gynaecology [7], mental health [8] and primary care [9]. Yet globally, clinicians often do not respond adequately to DVA [10]. In primary care, effective clinical management of common conditions (such as depression or unexplained pain) is not possible if a patient’s experience of abuse remains hidden [11]. IRIS is an evidence based innovative model of care that addresses this gap in healthcare provision and the suboptimal response to DVA in primary care [9].

The IRIS pragmatic cluster randomised controlled trial in 24 intervention and 24 control general practices, in two English cities, showed a three-fold difference in identification of women affected by DVA and a seven-fold difference in referral to specialist DVA services between control and IRIS practices respectively [9]. This was the first evidence that a system level intervention could improve the healthcare response to DVA, by increasing the referrals made of women affected by abuse, to an IRIS advocacy worker (the advocate-educator). A Cochrane review shows that brief advocacy may reduce abuse, improve mental health and quality of life, especially for less severe abuse and in pregnant women [12]. IRIS with its focus on offering women referral for specialist DVA advocacy was also estimated to be cost-effective [13]. Qualitative analysis nested within the original IRIS trial showed that women were positive about being asked about abuse by health professionals and contact with DVA advocates [14]. Health professionals viewed IRIS as an acceptable intervention but had a concern about the four hours’ length of training [15]. Trial results showed a wide variation in DVA identification and referral rates between IRIS practices and amongst clinicians within IRIS practices [9].

Based on the original trial, the IRIS model has been included as an example of best practice in multiple policy and guidance documents, including by NICE [2], the WHO [1], the UK government [16], the Chief Medical Officer [17] and the Home Office [18].

DVA’s health effects are more burdensome than hypertension, obesity, high cholesterol and smoking in women of reproductive age [19]. DVA is the top contributor to death, disability and illness in these women [20]; its management in clinical practice warrants much greater attention. Gynaecological and sexual health problems are the most prevalent and persistent physical health consequence of DVA [21]. Long-lasting mental health problems include depression, anxiety and post-traumatic stress disorder - the most prevalent mental health sequelae [22].

In the UK, since the recession of 2008/09, violent crime against women has increased [23]. Yet between 2008 and 2013, funding for specialist support services’ has decreased by a third [24], despite DVA costing an estimated £11 billion in lost economic output, social services, emotional and medical costs in 2012 [25].

The IRIS programme was developed as a primary health care contribution to a societal response to DVA, linking general practice to DVA services. The training, support and referral pathway is a complex intervention that enables clinicians to ask about DVA, recognise the DVA in a woman’s life, understand and be able to discuss with her that abuse’s significance to her health whilst providing excellent clinical care, taking the abuse into account and offering a referral to a named specialist within a DVA support service.

Despite the trial evidence, the national policy documents supporting IRIS, and the initial commissioning of IRIS in 34 areas, we do not know whether the programme is sustainable and effective when implemented outside the trial context. We need to determine whether IRIS and its original trial results can be replicated in general practice settings over the longer term. The UK Medical Research Council advises “…effects are likely to be smaller and more variable once the intervention becomes implemented more widely, and…long-term follow-up may be needed to determine whether short-term changes persist” [26].

## Methods

### Primary objective

1. To measure the effectiveness of IRIS alongside a comparator intervention, in general practice, both designed to improve the healthcare response to DVA in primary care.

### Secondary objectives

2. To understand the process of IRIS implementation, using a mixed methods approach, including survey and qualitative data.
3. To evaluate IRIS implementation, alongside IRIS’ sustainability, with in depth case studies of two different IRIS areas using interviews, participant observation and document analysis.

### Design

A four year observational, pragmatic, mixed methods, implementation MRC phase IV study [27]:

The principal design is a segmented regression analysis of interrupted time series (ITS) data (primarily, referrals received by DVA workers) from general practices that implemented the IRIS intervention and a comparator borough in which the general practices did not implement the IRIS intervention.

There are approximately 386,277 women, aged 16 years and above (patients), registered at the 140 general practices, in the four north-east London implementation borough sites (A, B, C & D), for which IRIS was commissioned; and approximately 77,464 women aged 16 years and above (patients), registered at the 61 general practices, in an adjacent comparator north-east London borough site (E). The comparator borough has not implemented the studied intervention (IRIS) but instead used an alternative DVA initiative, during the time period examined.

Qualitative research is carried out in parallel, including a concurrent embedded, mixed-method process evaluation of IRIS implementation and two in-depth, local, IRIS case studies with a sustainability focus.

All results are integrated, by considering the quantitative and the qualitative results alongside each other, checking that results coincide (for example, are areas with the highest incidence rate ratios for referrals received also the areas in which IRIS has the greatest training reach) whilst reflecting on discordant results, in order to increase understanding about IRIS implementation, sustainability and effectiveness.

### Setting

This implementation evaluation involves eight sites. Four of these are northeast London borough implementation sites (A, B, C & D) that commissioned IRIS within the study period. One is a comparator northeast London borough site (E) that did not commission IRIS but an alternative DVA initiative. One is an original IRIS trial intervention borough (F) that commissioned IRIS after the original trial. Additionally, an urban northern IRIS area (G) had an IRIS service started within the study period. The personnel involved included all staff at each general practice (clinical and administrative), DVA service providers’ staff and commissioners, including NHS clinical commissioning group staff (clinical leads for child/adult safeguarding and women’s health) and local council staff (concerned with local DVA strategy and public health).

### Population/ participants

Sites are invited to take part in this research due to their geographical location in the North Thames area of London, adjoining the original IRIS intervention trial site with a priori knowledge that areas are interested in IRIS commissioning. One IRIS site outside of London is included as a qualitative case study, as it fulfilled pre-specified inclusion criteria of this work (see Appendix A). IRIS targets women affected by DVA, either currently or historically, from a partner, ex-partner or an adult family member. The eligibility criteria to be included in this observational study are: female patients aged 16 and above, registered at a general practice, at the sites being studied. Women affected by DVA are identified by a clinician and offered a referral to the named IRIS advocate-educator (AE); or women can self-refer to IRIS if they see the publicity material displayed within a surgery.

### Intervention

IRIS is a general practice-based DVA training, support and referral programme for primary care staff. The theoretical framework of the training is based on educational outreach, adult learning theory and peer influence. It was developed using the MRC framework for complex interventions, to improve the primary care response to DVA. This involved steps for development, piloting and testing the intervention in a trial design followed by implementation in routine general practice [26].

The IRIS model consists of five core components:

1. Practice based DVA training, with two two-hour sessions for the whole practice team.
2. A local GP, interested in DVA, appointed as an IRIS clinical lead (CL) delivers clinical training alongside an IRIS AE.
3. A named DVA specialist – an IRIS AE - employed and based in a local DVA service, delivers training and also receives referrals from clinicians, responsible for a caseload of work, providing on-going support, refresher training and consultancy for the entire practice team, on a day to day basis when in the practice (preferably attending regular quarterly practice meetings), by phone and email.
4. The AE sees women affected by DVA, providing expert advocacy, including risk assessments, safety planning, emotional & mental health support, housing advice, referring, (e.g. to multi-agency risk assessment conferences (MARACs), child safeguarding services), support on injunctions & criminal justice system, signposting (e.g. to specialist DVA legal services) and accompanying to police or courts.
5. An electronic prompt, reminds clinicians to ask about DVA, considering its multiple dimensions [28] and a template within which to record DVA (encouraging clinicians to (i) assess the immediate safety of the woman and any children, (ii) offer referral, (iii) review within general practice). The electronic prompt is triggered by codes for health conditions or symptoms associated with DVA, such as fatigue, insomnia, anxiety and depression.

The AE is an experienced specialist support DVA worker, with previous training experience. The CL is interested in DVA as a health issue, preferably also with training experience. Both the IRIS AEs and CLs have completed the national IRIS Training for Trainers programme, delivered by IRISi staff,^1^ over three and two days respectively. The IRIS model is based on one full-time AE working with 25 general practices. The model is built upon partnership work, with primary care and specialist third sector agencies coming together to deliver services and promote work across the interdisciplinary gap. Further details about training (e.g. flowcharts showing simple referral pathways and various IRIS publicity materials to display in practices) are given in Additional file 1.

### Comparator intervention

The alternative DVA intervention delivered in the comparator site, E, also comprised a DVA education package. This involved training delivered away from general practices, training was delivered by those from an advocacy background (not clinicians), clinicians’ referrals were sent to a One Stop Shop (not named advocates), with no electronic prompt embedded within the electronic medical record, encouraging clinicians to ask about DVA when clinically relevant. The comparator site was not chosen at random. It was invited to participate in this research because it had declined to commission IRIS, but had commissioned an alternative DVA education package.

### Outcomes

#### Effectiveness outcomes

The primary outcome measure is the number of referrals received by DVA service providers from general practices. The denominator is the number of women aged 16 years and above within the practice. This is an evidence-based, intermediate outcome measure, on a causal pathway towards decreased DVA, and possibly better mental health and improved quality of life for women who are referred to specialist DVA services [12].

The secondary outcome measure is the number of women in whose medical records electronic DVA codes, representing DVA identification are used. The denominator is the number of women aged 16 years and above registered within each practice. Other pre-specified secondary outcome measures are the number of referrals received by MARACs from general practice, the type of contact and support offered to women by DVA advocates.

#### Process outcomes

Context for IRIS implementation, factors influencing IRIS implementation, local adaptations of IRIS and the reach of IRIS training is examined, at the northeast London IRIS sites - A, B, C, D and F.

#### Qualitative outcomes

Theoretically informed analysis of the features contributing to sustainable implementation of IRIS is conducted at sites B and G (see Appendix A).

### Data sources, collection and management

#### Quantitative data

We use historic and prospective data routinely collected from multiple sources:

1. DVA service providers: number of referrals received by their DVA workers, from general practices, with the date that each referral is received, the general practice from which it is received, the type of contact that is then made and the type of support required.
2. IRIS AEs: record data in referral spreadsheets, with each referral received, the referral date, the practice from which it is received, the contact type and support type, including whether a referral is made to a MARAC. They also record the dates of all IRIS training delivery, the number of practice staff that attend and who specifically delivers the training. These paper based records, are electronically entered on to spread sheets and shared with the researchers either directly or via IRISi.
3. General practices’ electronic medical records (EMR) for DVA codes used, each code with a date stamp and the general practice, at which it was used. EMR searches and data extraction are carried out remotely using the EMIS (Egton Medical Information Systems) web system located in the Centre for Primary Care and Public Health, Queen Mary, University of London.
4. Electronic searches conducted centrally extract the number of women aged 16 years and above registered at each general practice and the Index of Multiple Deprivation score.
5. Online anonymous questionnaire survey, incorporating an implementation measure based on Normalisation Process Theory [29] and administered via the Bristol Online Survey (BOS) tool (https://www.onlinesurveys.ac.uk/).
6. A five-item checklist assesses the fidelity of IRIS, identifying IRIS’ local adaptations.
  Is DVA training practice based?
  Is a local GP, the IRIS CL, delivering training alongside the AE?
  Is the IRIS AE, based in a local DVA service?
  Is the IRIS AE delivering training & receiving referrals?
  Is the electronic prompt & recording template, been activated?
7. Practice websites for the total number of practice staff employed. All contained a list of practice staff.

All extracted data are entered into the study database for statistical analysis.

#### Qualitative data

Qualitative data are derived from the free text comments generated by the online anonymous questionnaire survey (see above), participant observation, document analysis and interviews – semi-structured, using purposive samples of general practice staff (clinical and non-clinical), AEs, local stakeholders, including commissioners and IRIS service users. This work describes and understands rationale for any features unique to a particular site, capturing the views of professionals involved in implementing IRIS and patients who have been referred into the IRIS service.

### Data analysis

#### Quantitative data analysis

##### Sample size

The sample size was determined by simulation, using the SimSam package in Stata [30]. Simulations were of an analysis of monthly counts with a Poisson distribution. We assumed a typical practice had an average referral rate at baseline of 4.5 per 100,000 per month, based on data from the IRIS cluster randomised trial [9], with this rate varying between practices with a 95% normal range of 1.7 to 12.2 per 100,000 per month. We further assumed there were 3000 eligible women per practice, and 180 practices. We determined the number of months of data required to detect a doubling of the referral rate following the introduction of the intervention, with 90% power at the 5% significance level, assuming that different practices introduced the intervention at different times, uniformly over this period. Simulation showed that 17 months of data were required. In order to capture any seasonality in the referral rates we increased this to 24 months.

We use an interrupted time series segmented regression approach with a mixed effects Poisson regression model, to examine the effect of the IRIS intervention on the primary outcome measure (the number of referrals received by DVA service providers from general practices); and the secondary outcome measure (the number of first times that DVA identification electronic codes are used in medical notes by clinicians). We compare these outcome measures before and after the date of the first IRIS training session, taken from individual general practices (i.e. practice level data), from multiple general practices. This date of the first IRIS training session is taken as the date of IRIS implementation.

**Fig. 1:**
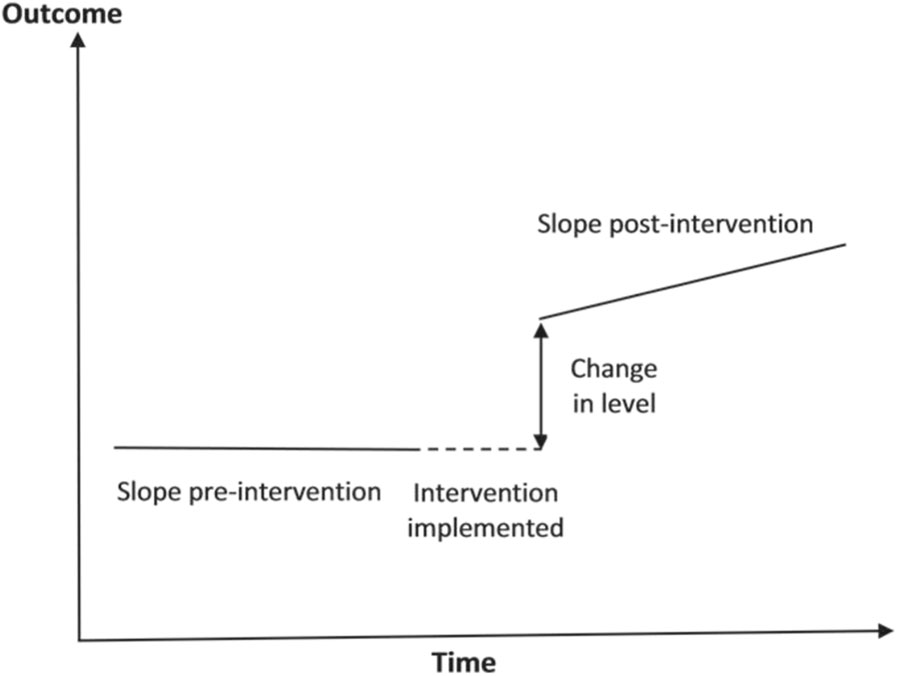
An interrupted time series segmented regression approach

Figure 1 depicts an interrupted time series segmented regression approach. Using this approach it is possible to examine whether there is a change in level immediately and/or a change in regression slope following the implementation of the intervention, i.e. a change in the gradient of the slope pre- and post-intervention.

For each practice the outcomes are defined as a daily frequency. The observations occurring on the day that the training is received are excluded, as it is not possible to say whether these occurred before or after training. Graphical displays of the data show average daily rates (per 1000 people) plotted against time centered on IRIS implementation. A moving average smoothing function, with equal weights, is applied to the data, with an unadjusted line of best fit added, before and after the training.

The ITS model includes a random effect of practice and fixed effects of training (pre or post in any given day in any given practice), the slope of the underlying time trend, the change in slope following the training, site (five London boroughs), and month and day of the week to allow for any seasonal effect of time. If for either outcome measure there is evidence of over-dispersion of the daily frequencies compared with a Poisson distribution then we model this with a random effect of day nested within the random effect of practice. We also adjust for the log-transformed number of women aged 16 years and above registered at that practice, every quarter, as an offset variable in the analysis. This allows us to control for the available population size at any one time. The analysis includes only those general practices that received IRIS training. We exclude practices where 50% or more of the list size data are missing, meaning we do not know the number of women aged 16 years and above registered at the practice. Regression coefficients are presented as incidence rate ratios. Data is analysed using the Stata V14 package (StataCorp LP, College Station, TX).

#### Sub-group analyses

A priori additional analyses include comparing the different implementation sites (A, B, C & D) to each other and the comparator site (E). The ITS analysis is run separately within each site. A forest plot is used to compare these individual analyses. Deprivation scores are used to check that deprivation does not confound the results.

#### Process evaluation data analysis

Survey data are exported from BOS to Stata V14 and analysed using descriptive statistics (means, standard deviations and frequencies) for the quantitative data.

#### Qualitative data analysis

Interview data are transcribed *verbatim* and coded thematically, using a mixture of inductive and deductive approaches. Theoretically informed analysis is conducted. Significant patterns are identified and the data examined for deviant cases.

## Knowledge mobilisation

We propose to present the findings from this study to key stakeholders at multiple local, national & international, non-academic and academic, conferences, meetings and workshops. We will also report our findings in academic and non-academic publications, sharing with national and international health policy fora, including contributions to the UK Government’s planned landmark Domestic Abuse Bill. The final IRIS ITS analysis results, supported with qualitative insights and web-based publicity will be presented to our local partners, including healthcare professionals, IRIS CLs, IRIS AEs, their managers and local third sector DVA host agencies, as well as local health care commissioners based in CCGs, Public Health, Local Authorities and the Police & crime commissioners whilst highlighting their role in integrating commissioning, using strategic partnerships.

## Discussion

This is the first interrupted time series analysis of an intervention to improve the health care response to domestic violence. The findings of this observational segmented regression interrupted time series analysis of GP IRIS implementation, outside a trial setting will characterise its suitability for national implementation in the United Kingdom. It will inform decisions about the future commissioning of IRIS in the UK. If the findings are positive, this will support the more widespread commissioning of IRIS despite the on-going constraints of austerity that are disproportionally reducing women’s services https://www.theguardian.com/world/2017/mar/09/women-bearing-86-of-austerity-burden-labour-research-reveals. Comparison of the implementation sites, using integrated quantitative and qualitative findings may help us understand the core components of IRIS that should be retained in the programme when commissioned locally or nationally so that the impact of IRIS is not diluted. If the findings are not positive then careful thought is required to consider what should be the next step, on what has been a difficult path, on the rough terrain of improving the healthcare response to DVA.

### Additional file

Additional file 1: IRIS publicity materials supplied. (ZIP 1802 kb)

## Appendix

### Pre-specified case study inclusion criteria

The following inclusion criteria are used to determine sites to be included as a case study:

- Sites not involved in the original IRIS trial
- Sites that had commissioned IRIS for more than two years
- Sites that are not anomalous in their delivery of IRIS - based on discussion with the IRIS Implementation team

## Abbreviations

AE: advocate-educator
BOS: Bristol Online Survey
CL: clinical lead
DVA: Domestic violence and abuse
EMIS: Egton Medical Information Systems – initial not used again
EMR: electronic medical record
IRIS: *I*dentification and *R*eferral to *I*mprove *S*afety
ITS: interrupted time series
MARAC: multi-agency risk assessment conferences
NICE: National Institute for Health and Care Excellence
QALY: quality-adjusted life years
WHO: World Health Organization

## Footnotes

1 **IRISi** – for further information see http://www.irisdomesticviolence.org.uk/iris/ & http://irisi.org
