# Supplementary material for: Improving the healthcare response to domestic violence and abuse in primary care: protocol for a mixed method evaluation of the implementation of a complex intervention
Source: BMC Public Health. 2018 Aug 3;18:971. doi: 10.1186/s12889-018-5865-z (PMC6091071; doi:10.1186/s12889-018-5865-z)
Supplement: Supplementary file 1 — IRIS publicity materials supplied. (ZIP 1802 kb) [file 12889_2018_5865_MOESM1_ESM.zip › PO_Corr/HiRes_IRIS_universal_cardR2.pdf]

You can get help to:

- Make your home safe
- Know your rights
- Build your confidence
- Plan a safe place to go
- Recover from the effects of domestic violence

Useful numbers

National Domestic Violence Helpline (freephone, 24 hr):
0808 2000 247

Men's Advice Line: 0808 801 0327

Respect: 0808 802 4040:
(if you are concerned about your own or someone else's violent behaviour)

In an emergency ALWAYS call 999

Domestic Violence Aware Practice

Are you a woman being hurt by someone in your family, are afraid of someone at home or are in a violent relationship?

Show us this card if you need help or talk to us here in private

IRIS Identification and Referral to Improve Safety

What is domestic violence?

Physical including being hit, kicked or attacked

Sexual having to be sexual when you don't want to

Emotional being called names, put down, made to feel bad or threatened

Financial when someone takes or controls your money

Isolation when someone controls who you see and when you go out

Everyone has the right to be safe at home

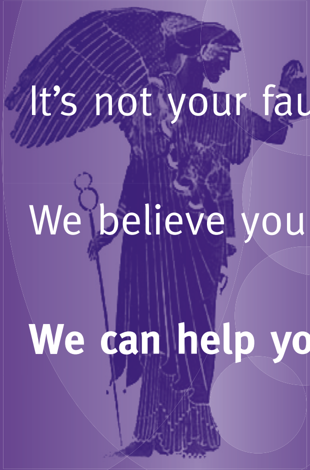

It's not your fault

We believe you

We can help you

We know that domestic violence does damage to health

If you choose to talk to us, we can:

- Support you
- Put you in touch with someone who can help you
