# Supplementary material for: Improving the healthcare response to domestic violence and abuse in primary care: protocol for a mixed method evaluation of the implementation of a complex intervention
Source: BMC Public Health. 2018 Aug 3;18:971. doi: 10.1186/s12889-018-5865-z (PMC6091071; doi:10.1186/s12889-018-5865-z)
Supplement: Supplementary file 1 — IRIS publicity materials supplied. (ZIP 1802 kb) [file 12889_2018_5865_MOESM1_ESM.zip › PO_Corr/HiRes_IRIS_universal_posterR2.pdf]

IRIS Identification and Referral to Improve Safety

Domestic Violence Aware Practice

If you are a woman being hurt by someone in your family, are afraid of someone at home or are in a violent relationship you can talk to doctors, nurses and other staff working here, in private.

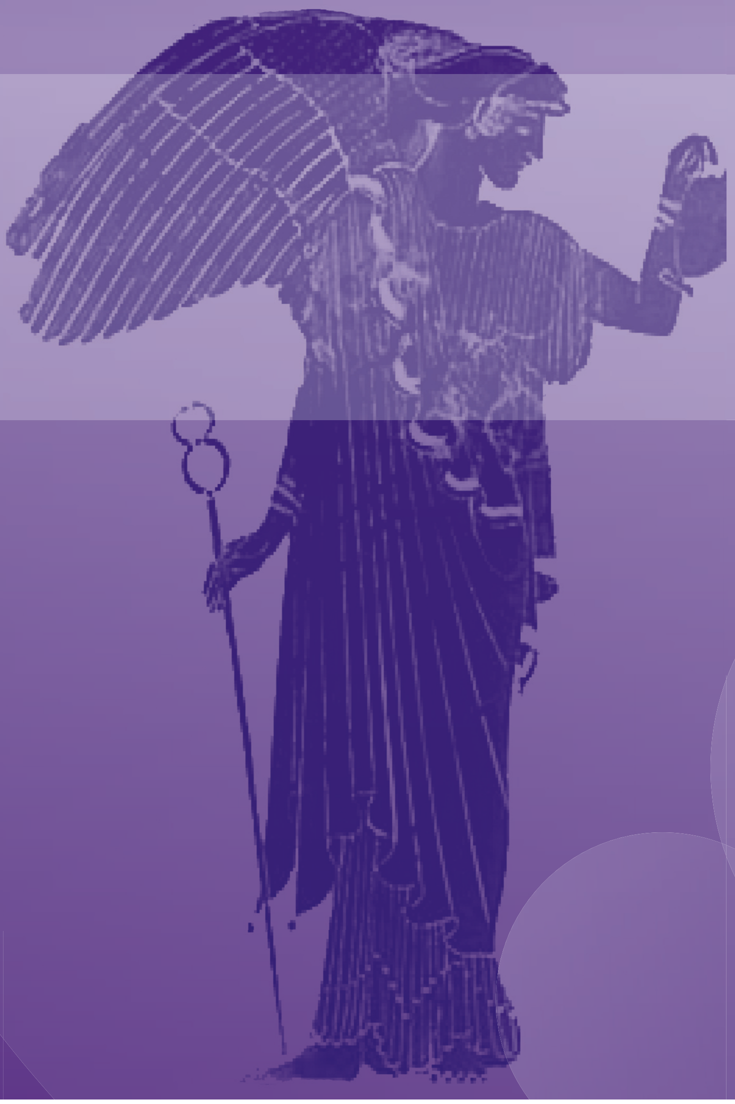

Or call the 24 hour
National Domestic Violence Helpline
on: freephone **0808 2000 247**

If you are a man who is a victim of domestic violence contact the Men's Advice Line on:

0808 801 0327

If you have been violent or are worried about your own behaviour, call Respect on:

0808 802 4040
